# Mini-Laparoscopic Repair of Apical Pelvic Organ Prolapse (POP) by Lateral Suspension with Mesh

**Published:** 2018-09

**Authors:** L Mereu, F Dalpra, E Terreno, R Pertile, S Angioni, S Tateo

**Affiliations:** Department of Obstetrics and Gynecology, Santa Chiara Hospital of Trento, Italy; Department of Clinical Epidemiology, Santa Chiara Hospital of Trento, Italy; Department of Obstetrics and Gynaecology, University of Cagliari, Italy.

**Keywords:** laparoscopic lateral suspension, learning curve, mini-laparoscopy, pelvic prolapse

## Abstract

**Background:**

The aim of the present study is to analyze the feasibility, safety and learning curve of Mini- Laparoscopic Lateral suspension (LLS) for the treatment of apical and anterior defects following pelvic organ prolapse.

**Methods:**

This is a cohort study on a retrospective series of 35 consecutive patients who underwent Mini-LLS for symptomatic POP between January 2014 and July 2016. All 35 patients were operated at the Gynaecological Unit in S. Chiara Hospital by two senior surgeons (S. Tateo and L. Mereu) and by a team with optimal skills in laparoscopic surgery. Patients were divided in two groups according to two different chronological phases: phase 1 identified the initial 12 cases, phase 2 the last 23 cases. We collected pre-, peri- and post-operative information to analyze the surgical outcomes and learning curve after Mini-LLS procedures.

**Results:**

The mean LLS-Overall Time (OT) was 107.6 min (range, 185- 63 min). None of the patients had intra-operative complications. No conversion to laparotomy was necessary. The mean post-operative hospital stay was 58 hours in total (SD +/-22). Only in 3 cases (8.6 %) post-operative grade I complications were observed. Recurrence of POP was observed in 3 cases (8.6 %) during a mean follow up of 18 months. The mean OT decreased with experience, in particular after the first 12 cases (phase 1: 113.54 minutes versus phase 2: 104.43 minutes). In consequence, the reduction of time per procedure was statistically significant considering the Cusum Time (CT) (P < .05).

**Conclusions:**

Mini-LLS with mesh is a safe and reproducible technique with good anatomical results, low complication rates and a short learning curve.

## Introduction

Pelvic organ prolapse (POP) is a highly prevalent condition requiring surgical treatment in 12-19% of cases (Barber et al., 2013; Smith et al., 2010). The primary objective of any POP surgery is to re-establish normal bladder, bowel and sexual function by resolving pelvic discomfort and bulge (Price et al., 2009). Different surgical approaches are described for the treatment of apical defects, with sacrocolpopexy considered as the gold standard for treatment (Barber et al., 2013; Ferreira et al. 2016), even if it can be associated with long operative time, a learning curve and with serious morbidity including vascular injuries, lesions of the superior hypogastric plexus, the right hypogastric nerve, spondylodiscitis and lumbar pain (Vieillefosse et al., 2015; Higgs et al., 2005; Veit-Rubin et al., 2016; Propst et al., 2014; Muffly et al., 2010).

Lateral suspension with mesh was described for the first time by Kapandji in 1968 and modified for a laparoscopic approach by Dubuisson in 1998. As such, it has been presented as an easier and safer alternative offering good results in terms of success rate and patient satisfaction (Veit-Rubin et al., 2017, Dubuisson et al., 2008; Simoncini et al., 2016; Veit-Rubin et al., 2016; 2017). The evolution of modern surgery is driven by the need to be minimally invasive to improve morbidity and cosmesis by reducing the incisional trauma, the number of ports or miniaturizing laparoscopic equipment (Bruhat et al., 1998; Wattiez et al., 1999). Mini-laparoscopy (M-LPS), with instruments that are ≤ 3mm in diameter has the advantage of requiring shorter operating times, patient position, and instrument configuration as conventional laparoscopy (Fanfani et al., 2012).

This paper presents the outcomes and learning curve of modified LLS performed with minilaparoscopic instruments allowing a reduced number of skin incisions. The primary goal of this study is to analyze the outcomes and learning curve of Mini- Laparoscopic Lateral Suspension (LLS) for the treatment of apical and anterior defects.

## Materials and methods

This is a retrospective cohort study on a series of 35 consecutive patients who underwent Mini-LLS for symptomatic POP between January 2014 and July 2016. All 35 patients were operated at the Gynaecological Unit in S. Chiara Hospital by two senior surgeons (S. Tateo and L. Mereu) and by a team with optimal skills in laparoscopic surgery. Patients with symptomatic stage 2 or greater (point C > -1 pelvic organ prolapse quantification POP-Q) apical prolapse (uterovaginal or vault prolapse) with or without anterior compartment prolapse (point Ba > -1 POP-Q) and without symptomatic or significant posterior compartment prolapse (point Bp >-1 POP-Q) were included in the analysis. Patients with apical +/- anterior and posterior prolapse underwent sacrocolpopexy. In 31 cases (88.6%) the apical prolapse concerned the uterus, in 3 cases (8.6%) the vaginal vault and in 1 case (2.8%) the cervix.

### Data collection

Informed consent to Mini-LLS was obtained from all patients in accordance with local and international law (WHO, 2001). Board approval was obtained from both the Ethical Committee of Azienda Provinciale Servizi Sanitari of Trento.

In pre-treatment evaluation, medical history collection, chest X-ray, pelvic ultrasound, physical examination, clinical evaluation of pelvic organ support assessed by the Pelvic Organ Prolapse Quantification Grading system (POP-Q), was included. Clinical characteristics of patients including age, BMI, menopausal hormonal status, sexual activity, parity, dyspareunia, bladder dysfunctions, co-morbidity and prior surgery for POP were recorded. Intra-operative parameters, including concomitant surgery, overall operating time (O-OT) and LLS operating time (LLS-OT), blood loss, conversion rate, post-operative pain. Time to discharge and recurrence were also recorded. O-OT was measured from the beginning of the skin incision to the completion of the skin closure, and LLS-OT was the O-OT minus the operative time needed to perform each concomitant surgery. The estimated blood loss was calculated as the difference in the total amount of suctioned and irrigated fluids.

Bladder dysfunctions were classified in urinary stress incontinence, urge incontinence and voiding dysfunction. Urodynamic evaluation was performed when required depending on the patients’ symptoms. Post-operative pain assessment was performed using a validated Numerical Rating Scale (NRS) at 6, 12 and 24 hours. Time to discharge was calculated in hours from the end of surgery to the moment of exit from the hospital. Post-operative parameters included short-term (within 30 days of the procedure) and long-term complications (more than 30 days after the procedure). Complications were measured by the Clavien-Dindo scale (Dindo et al., 2004). Systematic post-operative clinical and symptomatic evaluation was performed at 6 weeks, 6 months, one year and 2 years. Recurrence was defined as clinical POP greater than second stage or symptomatic POP.

### Surgical technique

**Figure 1:**
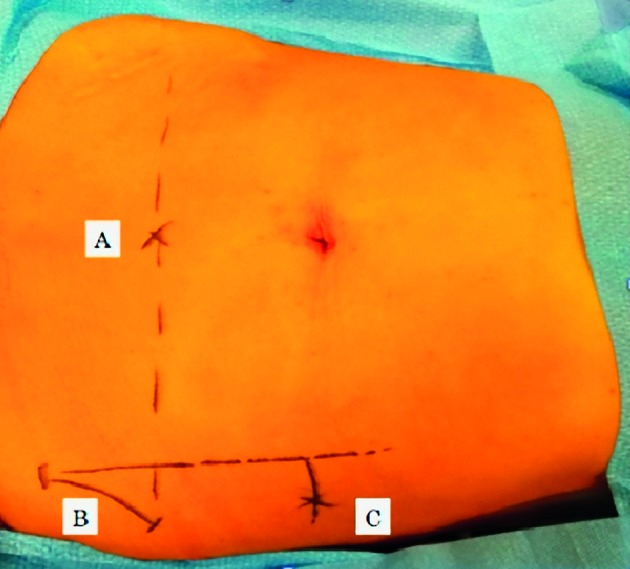
— Abdominal accesses. A: sovrapubic access; B: anterior superior iliaca spine; C: lateral access and lateral mesh suspension.

All patients were administered antibiotic prophylaxis (Cefoxitin 2g intravenously) and post-operative low molecular weight Enoxaparin (40 mg/day subcutaneously). Patients who underwent the procedure under general anaesthesia were placed in the semi-dorsal lithotomic position and then draped. The vaginal cavity was cleaned with a povidone iodine solution and a Foley catheter was placed in the bladder (routinely filled with 10 ml of indigo carmine dye). The surgical technique used was a modification of the LLS procedure described by Dubuisson, to minimize the dimensions and number of abdominal incisions (Veit-Rubin et al., 2017). The same technique can be used for uterine, isthmus or vaginal vault suspension. Pneumoperitoneum was induced with a Veress needle inserted through the umbilicus. A 10-mm trocar was introduced infra-umbilically and 3 mm operative trocars were introduced into suprapubic, left and right iliac areas and a laparoscopic forceps was introduced retroperitoneally through lateral incisions on both sides (2 cm above the iliac crest and 4 cm posterior to the longitudinal line of the anterior superior iliac spine) and oriented under the round ligament until it reached the corresponding free arm of the mesh (Figure 1). After the incision of the anterior peritoneum, a dissection of the bladder-vaginal space was performed. A T-shaped tetanized polypropylene mesh (TiLOOP ® ; “Prof. Dubuisson” pfm medical ag, Köln, Germany) with an anterior vaginal part of 6 cm in length and 5 cm in width and two lateral arms 3 cm in width and 18 cm in length each were used. The mesh was introduced through the 10 mm optical trocar, fashioned over the anterior vaginal wall (Figure 1) of the vaginal retractor and sutured to the vagina and uterine isthmus with about six sutures of 2.0 non absorbable suture thread (Ethibond Excel, Ethicon, division of Johnson & Johnson, Livingson, Scotland), avoiding passage through the vaginal mucosa. Following, a laparoscopic forceps was introduced retroperitoneally through the lateral incision and oriented under the round ligament until it reached the corresponding free arm of the mesh. The mesh was grasped and pulled out slowly laterally to obtain a satisfactory tension. The mesh was suspended without suture according to the “tension-free” repair considered satisfactory if the mesh is “horizontal” to the plane passing between the two iliac accesses (Figure 2). The mesh was then cut at the level of the skin. Then, the peritoneum was closed over the mesh to completely retroperitonealize the graft with barbed suture 2.0 (V-loc, Covidien, USA). A lateral attachment was provided by retroperitoneal fibrosis over the side arms.

**Figure 2:**
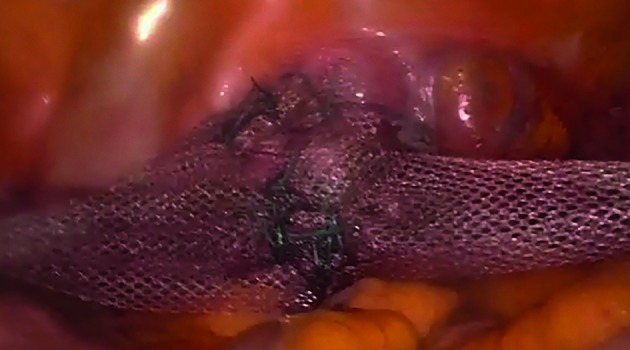
— Mesh lateral suspension.

### Cumulative Sum (CUSUM) analysis

We used the CUSUM technique fto quantify the learning curve for operative time reduction. ‘CUSUM Time’ was given by the sum of total differences between the individual data points and the mean of all the data points.

The CUSUM was used to assess the LLS-OT for all the 35 cases accounted. To calculate the CUSUM, the cases were ordered chronologically. For the first case, the CUSUM was the difference between the LLS-OT of the first case and the mean LLS-OT for all cases. The CUSUM of the second case was the previous case’s CUSUM added to the difference between the OT for the second case and the mean OT of all the cases. This calculation was continued up to the CUSUM calculation for the last case.

### Statistical analysis

Descriptive analyses included the calculation of observed frequencies (percentages) for each categorical variable, while means and standard deviations (SD) were computed for continuous variables (age, BMI, OT, CT and postoperative hospital stay). The Wilcoxon-Mann-Whitney test (a non-parametric statistical test) was used to compare the mean values of continuous variables between cases in phase 1 and 2.

The Fisher’s exact test was used to see if there was a significant difference between phase 1 and phase 2 for the following categorical variables: menopause, sexual activity, abdominal surgery, complications, surgeon A and surgeon B. The statistical significance level was p-value ≤.05 for unilateral probability in each test.

## Results

Between January 2014 and July 2016, 35 consecutive patients underwent Mini-LPS lateral suspension using a mesh to treat symptomatic anterior and apical prolapse. The predominant symptoms were prolapse-related symptoms such as a feeling of heaviness in the lower abdomen, a bulging sensation or a lump in the vagina, whether or not associated with an urge/stress of incontinence or voiding obstruction. The surgical indication was the central +/- anterior compartment prolapse: there were 8 (22.9 %) patients with point C > -1 and < 1 POP-Q and 27 (77.1 %) patients with point C > 1 POP-Q. In 31 cases (88.6%) the apical prolapse concerned the uterus, in 3 cases (8.6%) the vaginal vault and in 1 case (2.8%) the cervix (table I and table II). The median age of the patients was 53.5 years (SD ± 9.1) and the median BMI was 25 (SD ± 2.5). 65.7% of patients had a menopausal hormonal status and 82% were sexually active. 5 patients had preoperatively dyspareunia > 4. 42.9% of cases had had a prior abdominal surgery. Concomitant surgeries performed were: hysteroscopy in 6 cases, adhesiolysis in 3 cases, myomectomy in 1 case, correction of inguinal hernia in 1 case, bilateral salpingo-oophorectomy in 3 cases, bilateral salpingectomy in 1 case, transobturator vaginal tape (TOT) in 1 case. The mean LLS-OT was 107.6 min (range, 185.63 min). None of the patients had intraoperative complications. No conversion to laparotomy occurred. Mean blood loss was minimal in all cases (< 50 ml). The Foley catheter was removed in the morning after surgery and only one patient developed a urinary tract infection with incomplete bladder emptying requiring intermittent catheterization for 7 days after the surgery. All patients were successfully mobilized on the first day after the surgery. Postoperative analgesia included paracetamol 1 gr every 8 hours and ketorolac 30 mg every 12 hours during the first 24 hours. According to the NRS, no patients complained for pain ≥ 4 at 6, 12 and 24 hours. The mean post-operative hospital stay was 58 hours in total (SD ± 22). In 3 cases (8.6 %) there were post-operative grade I complications on the Clavien-Dindo scale: two patients felt lumbar pain (5.7%) and urinary tract infection was noted only in 1 case (2.8%) (Table III).

**Table I:** Pre- and postoperative POP-Q stage.

Stage (POP-Q)	Preoperative	Postoperative
Anterior compartment (cystocele)		
0	0	31
I	3	2
II	14	1
III	18	1
IV	0	0
Apical compartment (hysterocele, vaginal vault)		
0	0	33
I	4	1
II	18	0
III	13	1
IV	0	0
Posterior compartment (elythrocele, enterocele)		
0	12	26
I	20	4
II	3	4
III	0	1
IV	0	0

**Table II:** POP-related symptoms.

Symptoms	Preoperative n = 35 (%)	Postoperative n = 35 (%)
Vaginal bulge	35 (100)	2 (5.7)
Urge incontinence	11 (31.4)	1 (2.8)
Stress incontinence	1 (2.8)	0 (0)
Voiding obstruction	7 (20)	0 (0)

**Table III:** Intra and postoperative data.

Variables	Results
*O-OT, minutes (SD)	121.5 (43.2)
**LLS-OT, minutes (SD)	107.6 (29.0)
Concomitant Surgery, N (%)	15 (48.6)
Laparotomic Conversion, N (%)	0 (0.00)
Intraoperative Complications, N (%)	0 (0.00)
Postoperative hospital stay, hours (SD)	58 (22)
Postoperative Complications, N (%)	3 (8.6)
Recurrence of prolapsed, N (%)	3 (8.6)

* Overall operative time ** Laparoscopic Lateral Suspension operative time

The mean follow-up was 20 months with a range of 12-24 months. All patients underwent follow up at 6 weeks and 6 months, 32 women at 12 months and 28 patients at 24 months. Recurrence of POP was observed in 3 cases (8.6%). In one case, asymptomatic clinical rectocele, point Bp > 1 POPQ, occurred after 3 months, but it did not require any treatment. One case involved symptomatic recurrence after 20 months (point C and Ba > +1 POPQ, associated with urge incontinence and vaginal bulging) requiring surgical intervention (laparoscopic promontosacroplexy). In the third case, recurrence of anterior compartment prolapse (point Ba > -1 and <1) associated with persistent urgency occurred 1 month after surgery, but completely regressed after 6 months of vaginal treatment with oestriol and Kegel’s exercises (Kegel et al., 1948). No cases of erosion or extrusion were reported during the follow-up. From a functional standpoint, the main preoperative complaints (vaginal bulging, voiding obstruction and urinary incontinence) were completely resolved in 94.3% of patients (N=33). No de novo urgency symptoms were observed. The CUSUM learning curve (Figure 3) demonstrated two different phases: phase 1, identifying the first group (the initial 12 cases) and phase 2, identifying the second group (the last 23 cases). The mean LLS-OT decreased after the first 12 cases (phase 1: 113.54 minutes versus phase 2: 104.43 minutes). A comparison of various parameters between the two phases identified by CUSUM analysis is summarized in Table 4. In terms of demographic and clinical characteristics, recovery time and complications, no statistical difference was observed between the two phases. The time reduction was statistically significant considering the CT (P <.05).

**Figure 2:**
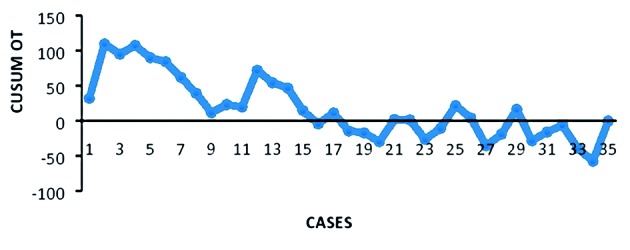
— CUSUM learning curve.

**Table IV:** CUSUM analysis.

Variables	Phase 1	Phase 2	P value
Age (year)	54.2	53.2	.365
∆ BMI	24.5	25.3	.291
Menopause (%)	58.3	69.6	.233
Sexual Activity (%)	91.7	78.3	.249
Abdominal Surgery (%)	25.0	52.2	.092
LLS-OT (min)	113.5	104.4	.279
CT (min)	61.9	- 5.6	<.0001
Postoperative hospital stay (hours)	54	60	.260
Postoperative Complications (%)	8.3	8.7	.729
Recurrence of prolapse (%)	16.7	4.3	.266
Surgeon A (%)	58.3	47.8	.408
Surgeon B (%)	41.7	52.2	

∆ Body Mass Index; ° Laparoscopic Lateral Suspension operative time; * Cusum Time

## Discussion

LLS with mesh are a feasible and safe technique promising long-term anatomic results and a high subjective cure rate of 82.7% at 7.5 years (Veit-Rubin et al., 2016). In the present study, the main surgical indications were anterior and/or central defects; the procedure is not indicated in case of predominant rectocele and/or pelvic floor insufficiency. The present surgical technique is a modification of the LLS technique described by Dubuisson et al. (2008) as far as the reducing dimension and number of abdominal incisions (four instead of six) are concerned. Lateral skin incisions were performed for both mini-laparoscopic instruments and extraperitoneal mesh positioning.

Dubuisson et al. (2008) described how the suspension axis of lateral meshes may lead to enterocoele, Pouch of Douglas hernia or descent of the upper part of the rectum. In the present series de novo posterior prolapse occurred only in one case (2.8%), not requiring surgical intervention. For this reason, we do not believe that an associated preventive posterior colpoperineorrhaphy or posterior mesh is required.

The possibility to preserve the uterus is an important aspect of this technique which reduces complications, operative time and is in line with recent publications, it stablishes a new concept for woman’s sexuality and fertility preservation (Zucchi et al., 2010). An overall achieved success rate of 94.3% is comparable to the success rate after sacrocolpopexy reported in previous studies (Barber et al., 2013; Gutman et al., 2013). We found an overall prolapse recurrence of 8.6% that is considerably low if compared to Dubuisson’s data (13%-17.8%) (Veit-Rubin et al., 2016; Dubuisson et al., 2008; Veit-Rubin et al., 201; Simoncini et al., 2016). In particular, posterior compartment relapse occurred in 2.8% of cases versus 7.3% and 11% of cases reported in the literature. Our data is in line with those reported by Simoncini et al. (2016) where the indication for lateral apical suspension was intermediate/advance apical and/or anterior prolapse. In case of significant posterior defect, concomitant posterior correction should be associated with the procedure, as suggested by Dubuisson et al. (2008) but sacrocolpopexy could also be considered.

One case (2.8%) of symptomatic anterior and apical recurrence occurred 20 months after surgery in a patient with a high BMI (30.4), requiring subsequent sacrocolpopexy. In our study, we found no cases of erosion or extrusion after a mean follow up of 18 months. However, mesh erosion is a recognized complication of mesh use, depending of several risk-factors: history of previous POP surgery, tobacco use, mesh type and position. Dällenbach et al. (2016) reported a mesh exposure and extrusion rate of 3.8% during LLS. In this study we used a T-shaped tetanized polypropylene mesh, which is reportedly less associated with the risk of erosion, if compared with Mersilene mesh (Dällenbach et al., 2016). The reason is that meshes with multifilament properties allow bacteria contamination but prevent the penetration of the host immune cells thus with an increased risk of infection (Klinge et al., 2002). A minimal invasive approach allows a reduction of postoperative pain and recovery time in the hospital. According to the NRS, no patients reported pain ≥ 4 during the postoperative hospital stay. In 2 cases (5.7%) lumbar pain was reported during the first visit after surgery (30 days) but a spontaneous remission was observed at the follow up (90 days). In the present series, hospital discharge occurred within a mean of 58 hours, which is half the time in comparison to data by Dubuisson et al. (2008) (4.4 days). This result could be explained by the reduced invasiveness of the procedure (number and dimension of scar incisions) and by the different selection of patients (women with posterior defect requiring posterior POP correction were included in Dubuisson’s study). Our mean overall operative time (OT) was 117 minutes, comparable with the results described by Simoncini at al. (2013) and considerably shorter than the 193 minutes reported by Dubuisson et al. (2013). Even though the two surgeons were confident in laparoscopy and in LLS, we found a statistical reduction of the OT after a certain number of cases, probably due to the fact that this modified technique of Mini-LLS needs an adequate learning curve. This prospective study analyzed the learning curve using the CUSUM technique. We reported not only an improvement in the average OT with increasing experience, as predicted, but also a statistically significant reduction of CT after the first 12 cases, which represent the initial phase of our learning curve.

A limitation to this preliminary analysis is the low number of patients accounted. Currently, we are performing a prospective study that will be able to provide immediate and long-term anatomic and functional results of LLS procedures for the treatment of apical +/- anterior prolapse.

## Conclusion

LLS may be considered an alternative choice for the treatment of symptomatic central prolapse, whether or not associated with anterior prolapse. It is characterized by a low rate of complications and prolapse recurrence, low postoperative pain and short recovery time. This study shows that even in experienced laparoscopic surgeons there is a short learning curve to perform lateral suspension by Mini-LPS. Further comparative studies between standard LLS and mini-LLS are necessary to evaluate the advantages and patient satisfaction.

## Disclosure statement:

The authors declare that they have no conflicts of interest and nothing to disclose.
